# Microbial community succession patterns and drivers of Luxiang-flavor Jiupei during long fermentation

**DOI:** 10.3389/fmicb.2023.1109719

**Published:** 2023-02-10

**Authors:** Xiaogang Liu, Dongna Ma, Chen Yang, Qianqian Yin, Shuangping Liu, Caihong Shen, Jian Mao

**Affiliations:** ^1^National Engineering Research Center of Cereal Fermentation and Food Biomanufacturing, State Key Laboratory of Food Science and Technology, School of Food Science and Technology, Jiangnan University, Wuxi, Jiangsu, China; ^2^Luzhou Laojiao Group Co. Ltd., Luzhou, Sichuan, China; ^3^Shaoxing Key Laboratory of Traditional Fermentation Food and Human Health, Jiangnan University (Shaoxing) Industrial Technology Research Institute, Shaoxing, Zhejiang, China; ^4^National Engineering Research Center of Huangjiu, Zhejiang Guyuelongshan Shaoxing Wine Co., Ltd., Shaoxing, Zhejiang, China; ^5^Jiangsu Provincial Engineering Research Center for Bioactive Product Processing, Jiangnan University, Wuxi, Jiangsu, China

**Keywords:** Jiupei, high-throughput sequencing, microbial community, driving factors, metaproteome

## Abstract

Luxiang-flavor Baijiu is the mainstream of Baijiu production and consumption in China, and the microbial composition has a great influence on the flavor and quality of Baijiu. In this study, we combined multi-omics sequencing technology to explore the microbial composition, dynamics and metabolite changes of Luxiang-flavor Jiupei during long fermentation periods. The results showed that based on the interaction between environmental constraints and microorganisms, Jiupei microorganisms formed different ecological niches and functional differentiation, which led to the formation of Jiupei stable core microorganisms. The bacteria were mainly *Lactobacillus* and *Acetobacter*, and the fungi were mainly *Kazachstani* and *Issatchenkia*. Most bacteria were negatively correlated with temperature, alcohol and acidity, and for the fungi, starch content, reducing sugar content and temperature had the most significant effects on community succession. Macroproteomic analysis revealed that *Lactobacillus jinshani* had the highest relative content; microbial composition, growth changes and functions were more similar in the pre-fermentation period (0–18 days); microorganisms stabilized in the late fermentation period (24–220 days). The metabolome analysis revealed that the metabolites of the Jiupei changed rapidly from 18 to 32 days of fermentation, with a significant increase in the relative content of amino acids, peptides and analogs and a significant decrease in the relative content of sugars; the metabolites of the Jiupei changed slowly from 32 to 220 days of fermentation, with a stabilization of the content of amino acids, peptides and analogs. This work provides insights into the microbial succession and microbial drivers during the long-term fermentation of Jiupei, which have potential implications for optimizing production and improving the flavor of Baijiu.

## 1. Introduction

Baijiu is one of the oldest distilled spirits and has a unique place in traditional Chinese culture (Wang, 2022). It is warmly welcomed by consumers worldwide for its distinctive and evocative flavors, which is mainly divided into three types Luzhou-flavor, Moutai-flavor, and light-flavor (Liu and Sun, 2018; Hong et al., 2020). Among them, Luzhou-flavor is characterized by aromatic and rich aroma, soft and strong fruit, and long aftertaste, occupying 70% of China’s total Baijiu production and playing a pivotal role in Baijiu industry (Fan and Qian, 2006). Luzhou-flavor Baijiu was produced by a cyclic solid-state fermentation process, the essence of which is a complex biochemical metabolic reaction that occurs in the solid, liquid, and gas phases of the brewing flora from the Jiupei, pit mud and the workshop environment (Zhou et al., 2021). The function of brewing flora can be simplified as a community metabolic process driven by microorganisms that convert substances such as starch into ethanol and flavor compounds by fermentation while saccharifying (Wang et al., 2020).

Jiupei (fermented grains) are the raw material for distillation of Baijiu, and the microorganisms in Jiupei mainly come from the Daqu and the mixed old Jiuzao (residue after Baijiu distillation), some of which can decompose the starch and cellulose in the grains, and the rest can use these decomposition products to produce fragrance and acid (Li et al., 2013; Xiang et al., 2013). The flavors in Baijiu are obtained by solid-state distillation of the fermentation metabolites of the microorganisms in the Jiupei at the end of fermentation. During this process, water vapor passes through the Jiupei and condenses, bringing out the flavor components. Therefore, the microorganisms in the Jiupei involved in fermentation are the main driving force in the formation of flavor substances, and the number, community structure, ecological succession and related metabolic activities of microorganisms in Jiupei directly affect the flavor quality of Baijiu (Yang et al., 2021). Jiang et al. (2020) analyzed the bacterial diversity of two Moutai-flavor Baijiu based on high-throughput sequencing, and the results showed high relative abundance of *Proteobacteria* and *Firmicutes*, and increasing comparable abundance of *Lactobacillus* and *Pseudomonas* with increasing fermentation time. Ma et al. (2020) studied Laobaigan-flavor Baijiu at different fermentation times and concluded that the microbiota formed by *Lactobacillus*, *Pediococcus*, *Weissella*, *Saccharomycopsis*, *Issatchenkia*, *Rhizopus*, *Trichosporon*, *Candida*, and *Aspergillus* contributed more to the trace components of Jiupei. Wang P. et al. (2018) studied the core microorganisms and flavor substances in Baijiu and found a highly significant correlation between the core microbiota and the evolution of the flavor profile.

Changes in the physical and chemical indexes of Jiupei during the fermentation process plays a significant role in Baijiu’s flavor. It not only affect the normal growth, reproduction, and metabolic activities of brewing microorganisms, but also influence the characteristics of brewing microbial populations and the formation of various aroma substances, which in turn have an impact on the sensory characteristics of Baijiu (Zhang M. et al., 2020). Therefore, studying the change pattern of physicochemical indexes of Jiupei during the fermentation of Luzhou-flavor Baijiu will help to better understand the mechanism of Luzhou-flavor brewing. At present, research on Jiupei microorganisms has focused on the metagenomic, and metaproteomics has been relatively slow (Chai et al., 2019a,b; Du et al., 2021). Because metaproteomics can reveal the nature of microbial function at the protein level, it is increasingly used in studies to assess the functional diversity of microbial communities (Yang et al., 2020). The lack of macroproteomics of Jiupei microorganisms has hindered the in-depth understanding of the fermentation mechanism of Baijiu. Therefore, we used multi-omics techniques, including amplicon sequencing, metaproteomics and metabolomics, combined with physicochemical indicators, to conduct a more in-depth analysis of Luzhou-flavor Jiupei. The study further investigated the changes of microorganisms and metabolites of fermented Jiupei during the long fermentation process, and analyzed the change patterns of flavor substances and microbial flora. The study provided basic data to support the analysis of the relationship between long fermentation time and original wine quality, and laid the foundation for an in-depth understanding of microbial community structures flora during the fermentation of Baijiu.

## 2. Materials and methods

### 2.1. Sample collection

We collected Jiupei from five Laojiao distilleries in Luzhou City, Sichuan Province for the long fermentation period, with sampling time points of 0, 2, 4, 6, 8, 10, 12, 14, 16, 18, 20, 22, 24, 26, 28, 30, 32, 36, 40, 44, 50, 56, 62, 68, 84, 100, 130, and 220 days. Natural fermentation was taken, with initial fermentation temperature, acidity and moisture content were 22°C, 1.9 mmol/10 g and 59%, respectively. Equal amounts of the Jiupei were collected from the upper and lower layers of the cellar and then mixed and labeled as one sample, and refrigerated at −80°C prior to sequencing.

### 2.2. Analysis of physicochemical indices

The determination of moisture content refers to “GB 5009.3–2016 Determination of Moisture in Foods.” For the determination of acidity, we refer to “Analysis and Detection of Wine Making.” The determination of liquefaction power refers to the literature of Sha Junxiang et al. For the determination method of glucoamylase activity, we refer to “General Test Method for Industrial Enzyme Preparations.” For the determination of acid protease activity, we refer to the “General Test Methods for Industrial Enzyme Preparations.”

### 2.3. DNA extraction, 16S rDNA amplicon sequencing

Genomic DNA was extracted using a FastDNA spin kit for soil according to the manufacturer’s instructions. The concentration and purity of DNA were determined by a NanoDrop 2000 UV–vis spectrophotometer (Thermo Scientific, Wilmington, United States) and stored at −80°C. To analyze the microbiology community, a target variable region of the small ribosomal subunit RNA gene was PCR-amplified with the KAPA HiFi Ready Mix (Kapa Biosystems CAT#KK2602), the full-length DNA was amplified with universal primers (Supplementary Table S1): then, the PCR product was purified by 0.6x AMPure PB Beads and constructed the library by using the SMRTbell Express Template Prep Kit 2.0 (PacBio) with damage repaired, end repaired, A-tailing, and ligated the sequencing adapters. The SMRTbell library was then purified by AMPure PB beads, and Agilent 2,100 Bioanalyzer (Agilent technologies, USA) was used to detect the size of library fragments. Sequencing was performed on a PacBio Sequel II instrument with Sequencing Primer V4 and Sequel II Binding Kit 2.1 in Grandomics. If the amplicon fragment ≥3 kb, the binding Kit is a 2.0 version (Bengtsson-Palme et al., 2013; Adedire et al., 2022).

The original FASTQ file was processed using QIIME software (Caporaso et al., 2010). After analyzing the sequencing data, the SILVA database was used to compare the 16S rRNA and ITS gene sequences to determine the taxonomic status of the corresponding microbes (Robeson et al., 2021). QIIME software was used to define sequence similarity >97% as an operational taxonomic unit (OTU), only OTUs containing at least five reads were considered to be valid in this study (Edgar, 2010). The Alpha diversity index was calculated to analyze species richness and uniformity in the samples; after which the Shannon index of the samples was determined to obtain the diversity of the community and the number of OTUs in the samples. Finally, the Beta diversity index was determined to analyze the heterogeneity of the community composition between samples.

### 2.4. Metaproteomics analysis

#### 2.4.1. Protein extraction and digestion

**Figure 1:**
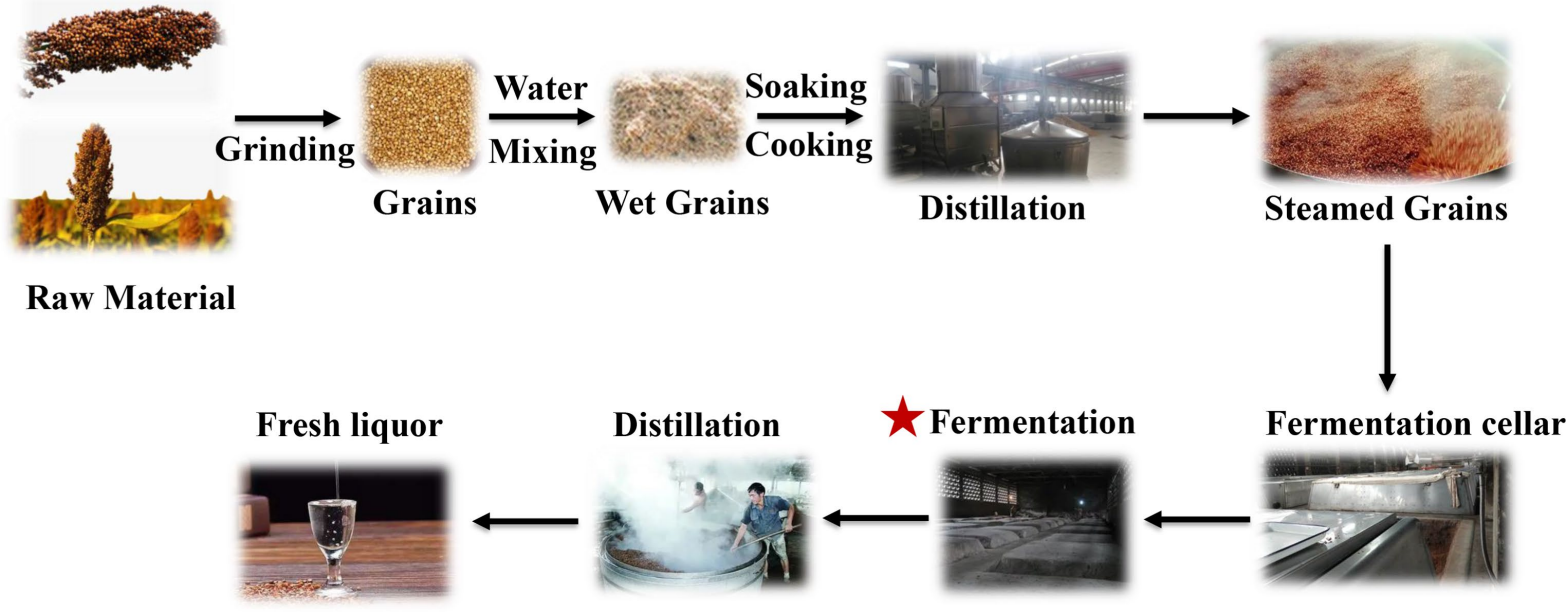
Jiupei production process diagram.

The collected Jiupei samples were powdered with liquid nitrogen in a sterile mortar. Each sample was accurately weighed 5 g, extracted with acetic acid–sodium acetate buffer (pH 4.6) for 6 h at 4°C, and then the supernatant was centrifuged to extract the supernatant, which was precipitated with 4 times the volume of trichloroacetic acid-acetone solution at −20°C overnight (Figure 1). After centrifugation, the precipitate was taken, followed by two washes with cold acetone, dried and concentrated by nitrogen blowing apparatus to obtain wine lees protein samples. The samples were stored at −80°C. 200 μg of protein was taken for digestion. Dithiothreitol was added to the protein sample to a final concentration of 10 mmol/l and reduced at 56°C for 1 h. Then 55 mmol/L iodoacetamide was added and the reaction was carried out at room temperature for 45 min. Four times the volume of pre-cooled acetone was added overnight at −20°C to precipitate the protein. After centrifugation, the precipitate was washed twice with 90% acetone and concentrated by nitrogen blowing, and then mixed with 1 ml of 50 mmol/L ammonium bicarbonate buffer. Next, trypsin was added in the ratio of protein: trypsin = 20:1, and the samples were incubated at 37°C for 12–16 h. The filtrate was collected and an amount of 0.1% trifluoroacetic acid solution was added, and then the samples were desalted (Zhang Y. et al., 2020).

#### 2.4.2. Peptide fractionation

The Shimadzu LC-20AB liquid phase system was used, and the separation column was a 5 μm 4.6 × 250mm Gemini C18 column for liquid phase separation of the sample. The dried peptide samples were reconstituted with mobile phase A (5% ACN pH 9.8) and injected, eluting at a flow rate of 1 ml/min by following gradients: 5% mobile phase B (95% ACN, pH 9.8) for 10 min, 5% to 35% mobile phase B for 40 min, 35% to 95% mobile phase B for 1 min, mobile phase B for 3 min, and 5% mobile phase B for 10 min. The elution peak was monitored at a wavelength of 214 nm and one component was collected per minute, and the samples were combined according to the chromatographic elution peak map to obtain 20 fractions, which were then freeze-dried (Rappsilber et al., 2007).

#### 2.4.3. HPLC

The dried peptide samples were reconstituted with mobile phase A (2% ACN, 0.1% FA), centrifuged at 20,000 g for 10 min, and the supernatant was taken for injection. Separation was performed by Thermo UltiMate 3,000 UHPLC. The sample was first enriched in trap column and desalted, and then entered a self-packed C18 column (75 μm internal diameter, 3 μm column size, 25 cm column length) and separated at a flow rate of 300 nl/min by the following effective gradient: 0 ~ 5 min, 5% mobile phase B (98% ACN, 0.1% FA); 5 ~ 45 min, mobile phase B linearly increased from 5% to 25%; 45 ~ 50 min, mobile phase B increased from 25% to 35%; 50 ~ 52 min, mobile phase B rose from 35% to 80%; 52 ~ 54 min, 80% mobile phase B; 54 ~ 60 min, 5% mobile phase B. The nanoliter liquid phase separation end was directly connected to the mass spectrometer (Erickson, 2000).

#### 2.4.4. Mass spectrometry detection

The peptides separated by liquid phase chromatography were ionized by a nanoESI source and then passed to a tandem mass spectrometer Q-Exactive HF X (Thermo Fisher Scientific, San Jose, CA) for DDA (Data Dependent Acquisition) mode detection. The main parameters were set: ion source voltage was set to 1.9 kV, MS1 scanning range was 350 ~ 1,500 m/z; the resolution was set to 60,000; MS2 starting m/z was fixed at 100; the resolution was 15,000. The ion screening conditions for MS2 fragmentation: are charged from 2+ to 6+, and the top 30 parent ions with a peak intensity exceeding 10,000. The ion fragmentation mode was HCD, and the fragment ions were detected in Orbitrap. The dynamic exclusion time was set to 30 s. The AGC was set to MS1 3E6, and MS2 1E5 (Chen and Li, 2019).

#### 2.4.5. Protein annotation and bioinformatics analysis

Tandem library search software was used to perform iterative library searches, and the reduced database files were imported into MaxQuant (1.5.3.30) for library search identification, followed by quantitative analysis based on information such as peptide peak intensities, peak areas, and liquid chromatographic retention times associated with the primary mass spectra, and a series of statistical analyses and quality control (Cox and Mann, 2008). Then, based on the identification results, functional annotation analysis of proteins such as Gene Ontology (GO) (Ashburner et al., 2000), Kyoto Encyclopedia of Genes and Genomes (KEGG), and species annotation analysis at each taxonomic level were performed (Kanehisa et al., 2014).

### 2.5. Metabolomic analysis

Untargeted metabolomic analysis to identify the differential metabolites between the different Jiupei samples was done using GC–MS and LC–MS. GC–MS analysis was performed using a 7890A gas chromatograph (Agilent) coupled to a PEGASUS HT mass selective detector (LECO). LC–MS analysis was performed on an Acquity UPLC system (Thermo Fisher Scientific) coupled with a Q Exactive HFX (Thermo Fisher Scientific). The detailed sample preparation and MS analysis methods are described in Text S1 (Sun et al., 2020).

### 2.6. Statistical analysis

We used Origin and SPSS software for data processing and analysis. Unweighted pair group method with principal coordinate analysis (PCoA) and analysis of similarities (ANOSIM) were carried out in R 4.0.5 (Chan, 2018). Redundancy analysis (RDA) was performed using CANOCO 4.5 software (He et al., 2019). The statistical significance of the difference between the means of samples was tested by one-way analysis of variance (ANOVA) with the Tukey *post hoc* test (McHugh, 2011). Gephi 0.9.2 was used for visualization of correlation analysis between microorganisms (Escandón et al., 2020).

## 3. Results

### 3.1. The succession of microbial communities during the fermentation of Jiupei

#### 3.1.1. Bacterial community structure characteristics

**Figure 2:**
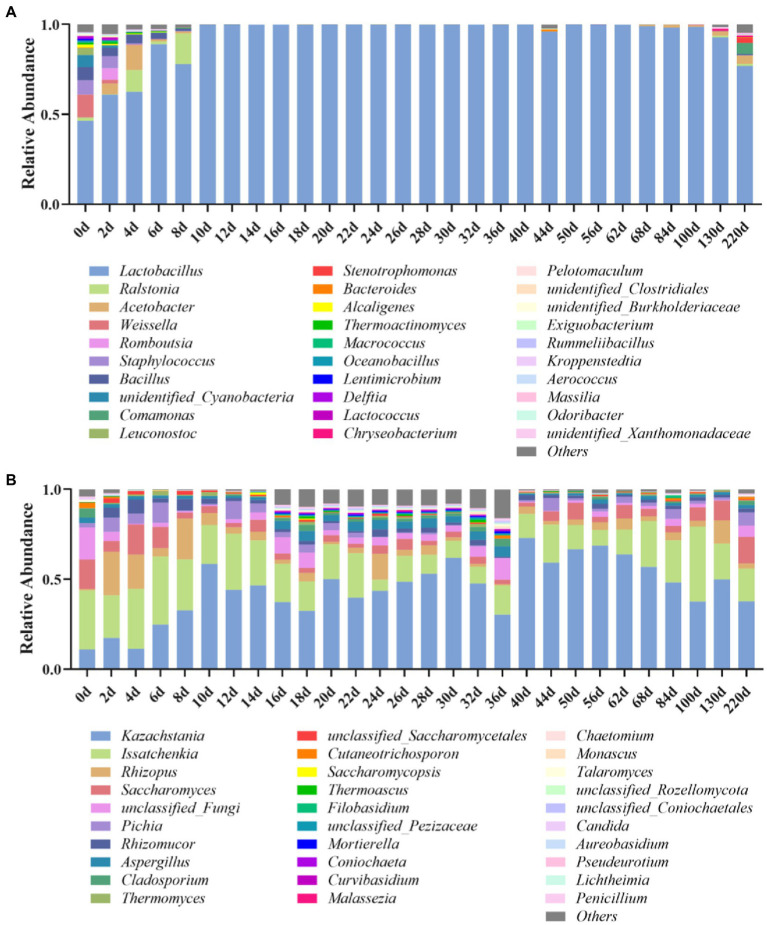
Bacterial **(A)** and fungal **(B)** microbial community compositions in Jiupei during fermentation process.

The microbial changes of Jiupei during fermentation were analyzed by 16S rDNA amplicon sequencing, and the results showed that the coverage of all samples were above 97%, indicating that the sequencing had sufficient depth and reliable throughput for subsequent analysis. At the genus level, a total of 336 bacterial species were detected in the Jiupei samples. Figure 2A shows the top 30 species in relative abundance, and the other species were merged into others. The top 10 relative abundances were *Lactobacillus*, *Ralstonia*, *Acetobacter*, *Weissella*, *Romboutsia*, *Staphylococcus*, *Bacillus*, unidentified_*Cyanobacteria*, *Comamonas*, and *Leuconostoc*. The relative abundance of *Lactobacillus* did not change much before entering the cellar for 4 days of fermentation. After 6 days of fermentation, the relative abundance of *Lactobacillus* increased rapidly, reaching more than 99%, becoming the dominant genus. After 100 days of fermentation, the relative abundance of *Lactobacillus* showed a downward trend again, and by 220 days of fermentation, *Lactobacillus* decreased by 22.2%. The relative abundance of *Lactobacillus*, which belongs to lactic acid bacteria, increased greatly in the sample as the fermentation progresses, and became the dominant bacteria in the sample. Various *Lactobacillus* occupy an absolute dominant position in the late stage of brewing and fermentation of Luzhou-flavor baijiu, which affects the flavor and quality of Luzhou-flavor baijiu. The *Weissella* genus accounted for a large proportion (12.6%) before entering the pit, and its proportion decreased continuously in the first 2 days of fermentation, and the relative abundance was less than 1%. *Ralstonia* increased in the first 8 days, and then decreased. The relative abundance of *Acetobacter* first increased and then decreased at 8 days before fermentation, and increased again after 130 days. The relative abundance of *Bacillus* decreased continuously 8 days before fermentation.

#### 3.1.2. Fungal community structure characteristics

At the genus level, a total of 654 genus fungal species were detected in the Luzhou-flavor Jiupei samples. Figure 2B shows the top 30 species in relative abundance, and other species were merged into others. It can be seen that the top 10 genera in relative abundance were *Kazachstania*, *Issatchenkia*, *Rhizopus*, *Saccharomyces*, *Pichia*, *Rhizomucor*, *Aspergilus*, *Cladosporium*, and *Thermomyces*. Before entering the cellar, the genus *Issaccharomyces* was the absolute dominant genus, and the genus *Kazachstania* was the absolute dominant fungal genus in the later stage of fermentation. The relative abundance of *Rhizopus* increased continuously in the first 4 days of fermentation, and the relative abundance of *Rhizopus* and *Aspergillus* decreased with the prolongation of fermentation time. The relative abundance of *Issatchenkia* increased after 6 days of fermentation and then decreased with the progress of fermentation. The relative abundance of *Saccharomyces* was increasing.

From the perspective of the succession of the fermentation community structure of Jiupei, the bacterial community was dominated by *Lactobacillus*, *Weissella* and *Bacillus*; the succession direction of the fungal community structure was *Issatchenkia*, *Saccharomyces*, *Rhizopus*, and *Aspergillus* were dominated, with *Kazachstania*, *Saccharomyces* and *Pichia* as the main substitutes.

### 3.2. Changes of physiochemical indicators during Jiupei fermentation

**Figure 3:**
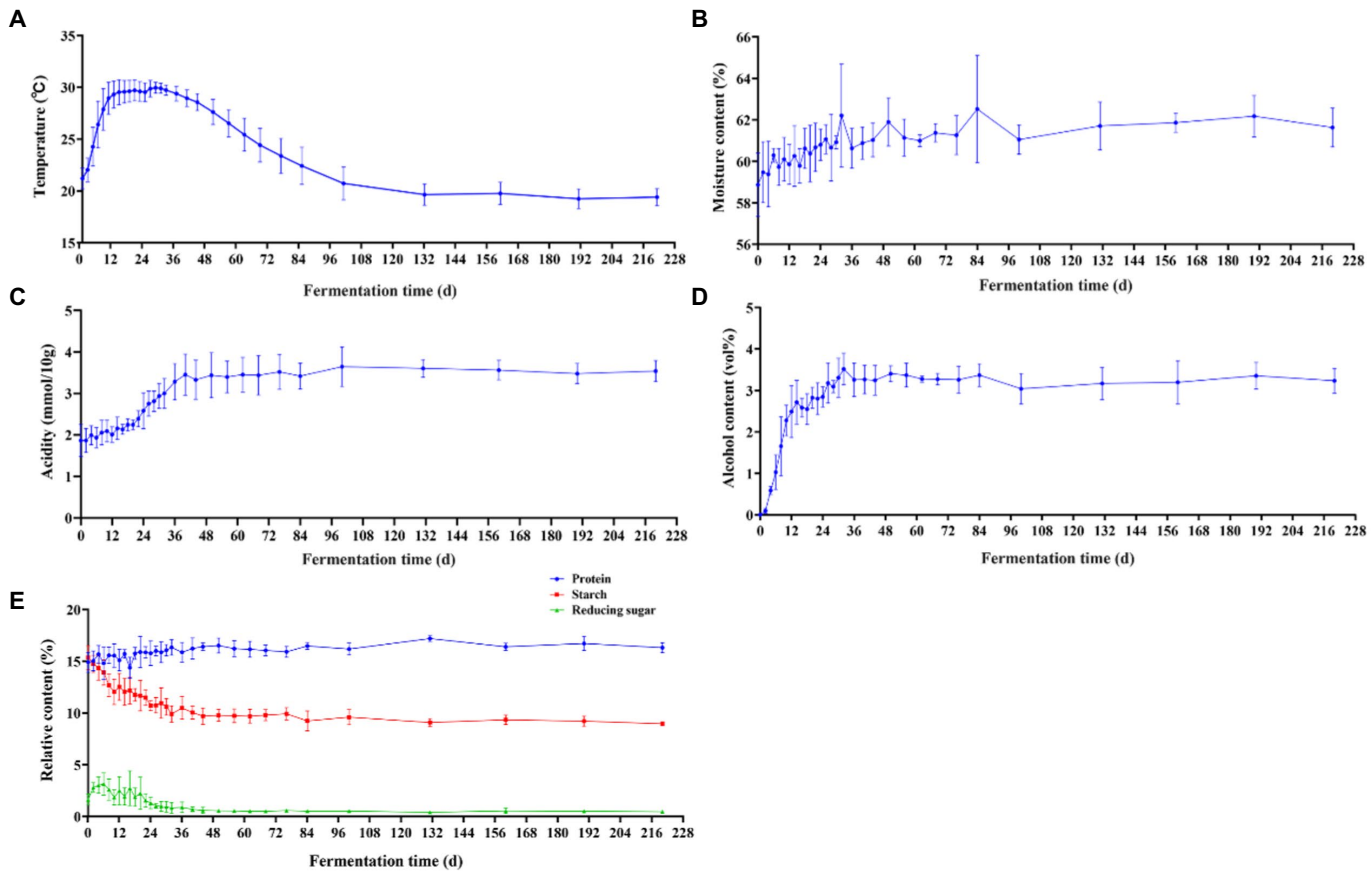
Changes in physicochemical parameters in Jiupei during fermentation process. **(A)** Temperature. **(B)** Moisture content. **(C)** Alcohol content. **(D)** Acidity. **(E)** Protein, starch, and reducing sugar content.

During the natural fermentation of Baijiu, the diversity and structure of the microbial community are influenced by a number of environmental factors, such as temperature, acidity, moisture and ethanol (Song et al., 2017; Wang X. et al., 2018; Tan et al., 2019). Temperature is the most common fermentation parameter in Baijiu research and affects flavor by influencing the microbial metabolites and content in the fermentation cellar (Zhang et al., 2021; Liu et al., 2022). Therefore, we investigated the temperature of the jiupei fermentation process. From Figure 3A, it can be seen that the fermentation process in Jiupei can be initially divided into four stages depending on the temperature. From 0 to 12 days, the temperature continued to rise, from an initial 21.20 ± 1.02°C rapidly raised to 28.94 ± 1.53°C. On the 12–34 days, the temperature fluctuated in the range of 29.30°C–29.94°C, and then entered the cooling stage, which was basically close to room temperature. On the 100th day, it reached 20.72 ± 1.58°C and then the temperature remained essentially constant. Overall, the parallel fluctuations in temperature were small, and the temperature change curve can be summarized as “slowly rising at the front, rising in the middle, and falling slowly at the back.”

It can be found that the moisture content, alcohol content and acidity of Jiupei varied greatly in the pre-fermentation period, and then presented a relatively stable state throughout the fermentation period. During the fermentation process, the moisture content increased continuously with time, and finally stabilized at 61.63 ± 0.94% (Figure 3B). During the pre-fermentation period (0–10 days), the alcohol content increased rapidly, slowly increased after 10 days, and reached the highest point of 3.52% ± 0.38% on the 32 days. After 36 days, the alcohol content of the fermented grains became flat (Figure 3C). From 0 to 36 days, the acidity increased from 1.87 ± 0.39 (mmol/10 g) to 3.45 ± 0.49 (mmol/10 g), the increase rate was slow in 0–10 days, and the increase rate was fast in 10–36 days, and then the acidity of fermented grains remained basically unchanged, the acidity at the end of fermentation was 3.54 ± 0.25 (mmol/10 g) (Figure 3D). Figure 3E showed the changing trend of protein, starch and reducing sugar contents during the fermentation of fermented Jiupei. It can be seen that the fermentation parameters were significantly different at different time points. The protein content in the fermentation process showed a trend of first increasing and then leveling off, and the protein content of Jiupei in the cellar was 14.88 ± 0.97%. At 130 days, the protein content reached the highest point of 17.18 ± 0.28%. The starch content decreased from 15.35 ± 1.15% to 8.95 ± 0.19%, with a rapid decrease from 0 to 12 days, and then a slow decrease from 12 to 36 days. The reducing sugar content increased slowly from 1.63 ± 0.54% to 3.14 ± 1.06% on the 0–6 days, and then decreased slowly, and the reducing sugar content was 0.46 ± 0.09% in the later stage of fermentation. Our results show that the 0–36 days were an important period for substrate (reducing sugar) utilization and product accumulation.

### 3.3. Driving factors of microbial communities during fermentation of Jiupei

**Figure 4:**
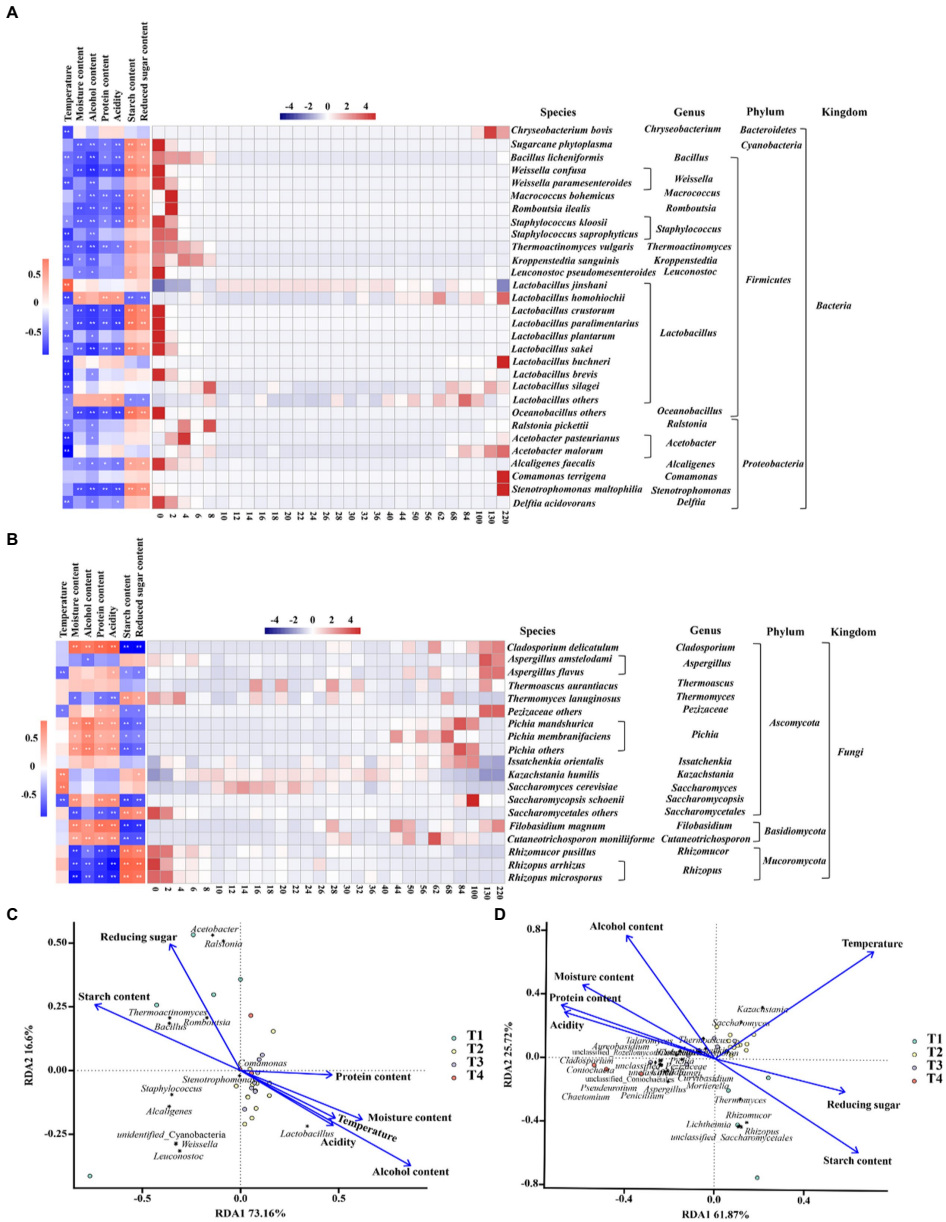
Changes in microbial community succession during the fermentation of Jiupei**. (A)** Community succession at the bacterial species level and its correlation with physicochemical parameters. **(B)** Community succession at the fungal species level and its correlation with physicochemical parameters. **(C)** The driving force in the fermentation process of bacterial of Jiupei. **(D)** The driving force in the fermentation process of fungal of Jiupei.

We analyzed the driving forces of microbial community succession, including biotic and abiotic factors. During fermentation, temperature, moisture content, alcoholic content, acidity, and protein, starch, and reducing sugar content significant differences throughout the fermentation process, and some of these environmental variables were associated with changes in community dynamics. As shown in Figure 4, the trends of the community at the species level were related to its adaptability to environmental conditions, indicating a gradual stabilization of the microbial community structure under the stress of various environments. For example, most of the bacteria were negatively correlated with temperature, alcoholic content and acidity, and *Lactobacillus homohiochii* was positively correlated with protein content. *Rhizopus microsporus*, *Rhizopus arrhizus* and *Rhizomucor pusillus* were negatively correlated with moisture content, alcoholic content, protein content, and acidity, and positively correlated with starch content and reducing sugar content (Figure 4A). In contrast, *Pichia mandshurica*, *Pichia membranefaciens*, *Pichia others*, *Filobasidium magnum*, *Cutaneotrichosporon moniliiforme,* and *Cladosporium delicatulum* were positively correlated with alcohol content, acidity and protein content related (Figure 4B).

The RDA results shown that the structural succession of the Jiupei bacterial community were highly influenced by the alcoholic content (Figure 4C). In the pre-fermentation, starch content was the main driving force for the succession of the bacterial community, and as fermentation progressed, alcoholic content, moisture content, and temperature became the main driving forces, which had a significant effect on *Lactobacillus*. The extremely small angles between alcohol and acidity, temperature and moisture content, implying a indicating a significant synergistic drive between them. From Figure 4D, we found that starch content and reducing sugar content were the main driving forces from 0 to 10 days of fermentation, while temperature had the most significant effect on fungal community succession from 10 to 36 days. *Rhizomucor*, *Rhizopus* and *Thermomyces* were positively correlated with starch and reducing sugar content in initial stage. The main microorganisms significantly affected by temperature were *Kazachstania*, *Saccharomyces*, *Thermoascus*, *Issatchenkia*, and *Cutaneotrichosporon*.

### 3.4. Protein and protein peptides characteristics during Jiupei fermentation

#### 3.4.1. Identification of microbial protein peptides and proteins

In this study, mass spectrometry (MS) data were collected for 39 microbial samples, and the statistics of spectra, peptides and protein for each sample were shown in Supplementary Table S2. Principal component analysis showed that there was an obvious succession trend in the fermentation process of Jiupei, which can be divided into three regions. The 0 day after the start of fermentation was located in the lower left corner, and the 4–18 days was the vigorous fermentation period, located in the upper left corner, and entered the stable fermentation period after 24 days (Supplementary Figure S1). The Pearson correlation clustering also revealed that the protein species were more consistent from 0 to 18 days and from 18 to 220 days (Supplementary Figure S2). After a series of quality control, a total of 1,525 proteins were identified from the microbial proteins obtained from different sampling points during the fermentation of Jiupei, and 646 proteins were distributed in the range of 10–20 kDa, with the highest content (Supplementary Figure S3).

#### 3.4.2. Protein species composition analysis

**Figure 5:**
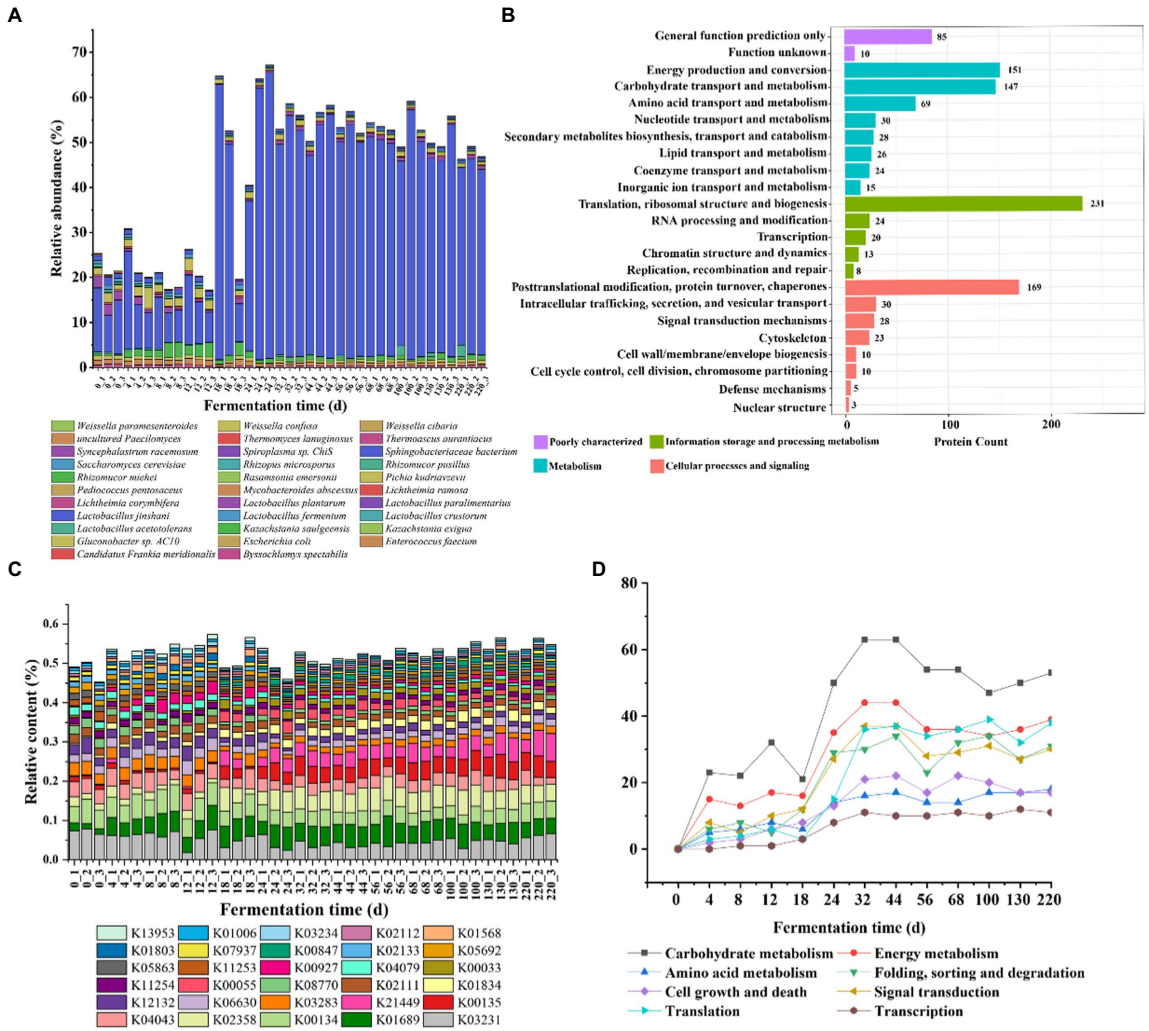
Species composition and functional annotation in Jiupei during fermentation process. **(A)** Microbial composition in Jiupei during fermentation process. **(B)** KOG function annotated histogram. **(C)** KO community structure function annotation. **(D)** Changes in the number of major pathways during fermentation.

We classified the species based on the comparison with the database, and the results showed that a total of 709 proteins were annotated to the species level, accounting for 46.49% (Supplementary Table S3). The proteins with higher relative abundance throughout the fermentation process were mainly derived from the top 10 species with the highest abundance in each sample. The relative abundance of *Lactobacillus jinshani* was the highest, and the relative content remained at 10% to 20% in 0–17 days of fermentation, and reached a peak in 18–24 days, accounting for about 50% of the total content, the abundance decreased slightly and remained stable during the 25–220 days. The relative abundance of *Kazachstania saulgeensis*, *Pichia kudriavzevii*, *Sphingobacteriaceae bacterium*, *Saccharomyces cerevisiae*, *Lactobacillus plantarum* in 0–220 days was greater than that of 25–220 days (Figure 5A).

#### 3.4.3. Prediction of proteins function

Subsequently, the public databases including GO, KEGG, KOG, CAZy and Swiss-Port were annotated to elaborate the functions of the identified proteins. Among them, 82.59% have GO term classification, GO biological process was annotated to 74.91%, GO molecular function was annotated to 79.64%, GO molecular composition was annotated to 52.17%; Approximately 66.29% of proteins have orthologous groups in KOG, 9.26% have CAZy, 73.39% could be mapped to known biological pathways, and Swissprot was annotated to 86.33% in total (Supplementary Table S4). In the KOG database, proteins were divided into 23 entries, with the largest number of 231 corresponding to translation, ribosome structure and biogenesis, implying that there was a large amount of fermented grains in the fermentation process (Figure 5B). Furthermore, the top five functions in terms of protein were posttranslational modification, protein turnover, chaperones, energy production and conversion, carbohydrate transport and metabolism. It showed that microbial proteins mainly perform information storage and processing and metabolism-related functions during fermentation (Figure 5B). Interestingly, it was found that most proteins changed little during fermentation, while proteins performing carbohydrate transport and metabolism and amino acid transport and metabolism increased significantly on the fourth day; proteins produced by membrane biogenesis increased significantly and to a greater extent on the 18th day, indicating that gene expression and metabolism of microorganisms in the system changed significantly on the fourth day of fermented grains (Supplementary Figure S4). Counting the abundance of different functions in each sample, it can be found that succinate-semialdehyde dehydrogenase, elongation factor Tu, trimeric autotransporter adhesin, which were less abundant before 18 days of fermentation and increased significantly after 18 days; serine-type D-Ala-D-Ala carboxypeptidase, actin beta, and L-cystine transport system substrate binding protein showed opposite changes in abundance (Figure 5C). In order to explore the effect of microbial gene expression on Jiupei, KEGG pathway annotation was performed on the detected microbial proteins. The pathways involved in these microbial proteins mainly include carbohydrate metabolism, energy metabolism, translation, signal transduction and folding, sorting and degradation (Supplementary Figure S5). It can be seen that the content gradually increased with the prolongation of fermentation time, and the most rapid growth occurred at 18 days, decreased slightly, and stabilized after 32 days. The obvious changes in the number of proteins involved in these pathways suggested that fermentation for 18 days might be a critical time point for the role of microorganisms in the fermentation process (Figure 5D). A total of 47 glycoside hydrolases (GHs), 36 glycosyltransferases (GTs), 2 polysaccharide lyases (PLs), 12 carbohydrate hydrolases (CEs), 26 carbohydrate binding enzymes (CBMs) and 18 accessory module enzymes (AAs) were annotated in the CAZy database (Supplementary Figure S6A). Further analysis revealed that the contents of *CBM18*, *GH135*, *GH23* and *GT27* showed a significant increase at 18 days of fermentation, while *CBM21* and *CE16* showed a significant decrease (Supplementary Figure S6B).

### 3.5. Metabolomics study on fermentation process of Jiupei during long fermentation

#### 3.5.1. Principal component analysis

**Figure 6:**
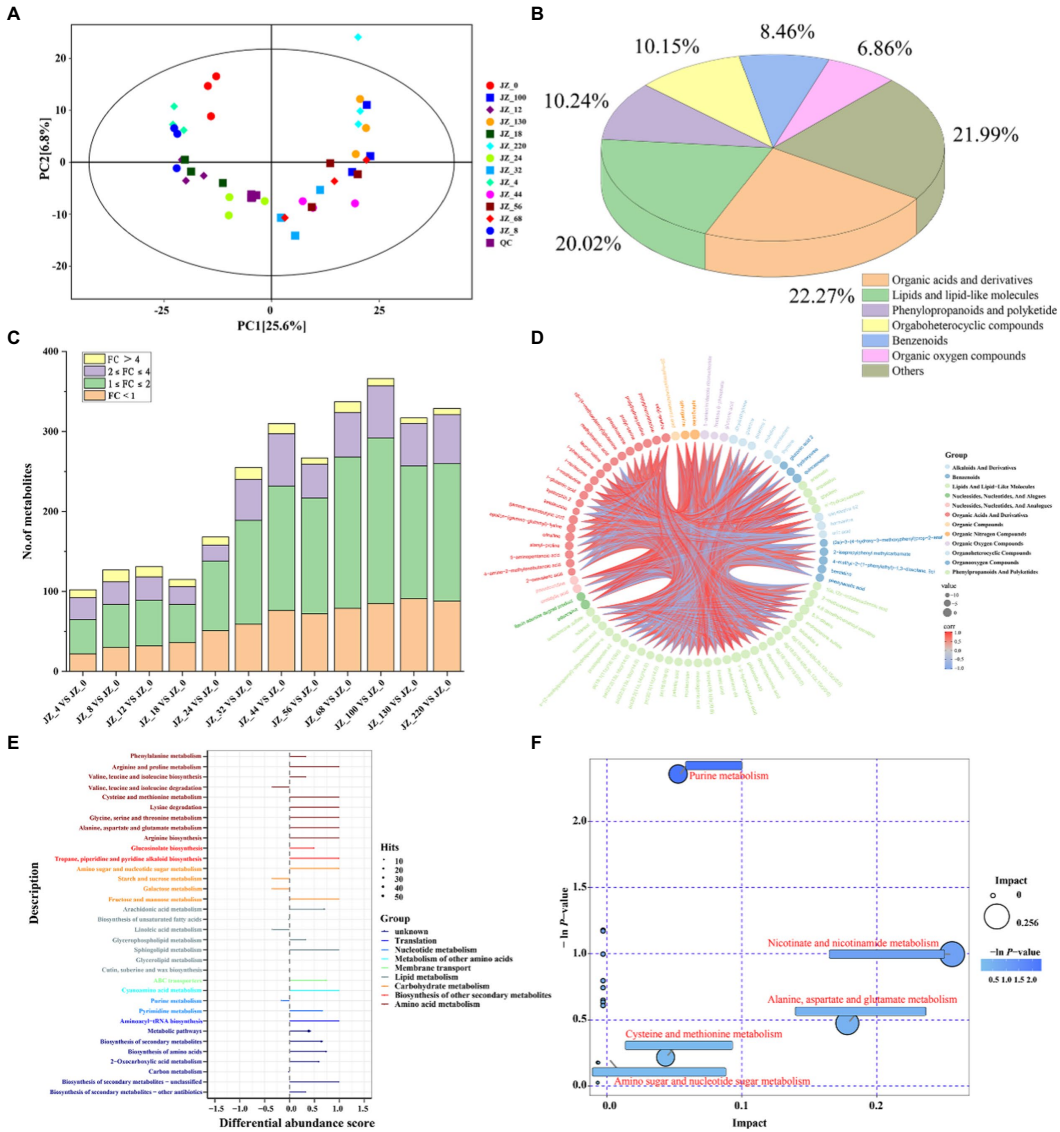
Metabolome analysis in Jiupei during fermentation process. **(A)** PCA score plot in Jiupei during fermentation process. **(B)** Classification of fermented Jiupei metabolites. **(C)** Differential metabolite species compared at different fermentation time points and the 0th day of fermentation. **(D)** Differential metabolite analysis of Z_18 *VS* JZ_0. **(E)** KEGG pathway analysis for differential metabolites. **(F)** Pathway bubble diagram of differential metabolites.

We used LC–MS and GC–MS analysis to further investigate the differences and dynamic changes of metabolites during the fermentation of Jiupei, and sampling time points were 0, 4, 8, 12, 18, 24, 32, 44, 56, 68, 100, 130, 220 days, numbered JZ_0, JZ_4 … JZ_220. PCA analysis reflects the clustering of metabolites in the fermentation process; the closer the metabolic levels, the closer the points on the score plot. PCA was performed based on the obtained data. As shown in PCA score plots (Figure 6A), QC samples were clustered together, indicating good inter-sample repeatability and credible experimental data for the in-depth analysis. The first principal component (PC1) accounted for 25.6% of the variance, and the second principal component (PC2) accounted for 6.8% of the variance. The metabolites during the fermentation of Jiupei changed gradually, and the samples at different times were arranged chronologically on PC1, which also indicated that the changes of the groups had a tendency to move in time, which also implied that the fermentation of Jiupei caused the chemical composition to change continuously.

#### 3.5.2. Metabolite identification

In the untargeted metabolomic analysis, 1,064 metabolites were co-qualified during the fermentation of the Jiupei (Figure 6B), among which organic acids and derivatives were the most types, accounting for 22.27%, followed by lipids and lipid-like molecules (20.02%), phenylopropanoids and polyketide (10.24%), orgaboheterocyclic compounds (10.15%), benzenoids (8.46%) and Organic oxygen compounds (6.86%). The results also showed that a total of 200 amino acids, peptides and their similar substances were identified, mainly including proline, leucine, ornithine, and lysine, the content of which increased rapidly at 18 days of fermentation, reached a maximum at 44 days and remained dynamically stable at the later stage of fermentation (Figure 6C).

#### 3.5.3. Differential expressed metabolites analysis

We used the OPLS-DA model to extract information on the differences of metabolites in Juppei samples with different fermentation times, using JZ_0 as the control group. The differentially expressed metabolites (DEMs) identified if VIP > 1 and *p*-value < 0.05. The results showed that there were fewer DEMs (100–200) between the first 24 days of fermentation and 0 day, and more than 200 DEMs between the first 32 days of fermentation and 0 day. Combined with PCA score plot (Figure 6A), it could be found that samples from fermentation 4, 8, 12, and 18 days and samples from 32, 44, 56, 68, 100, 130, and 220 days cannot be well separated, while on the 24 days of fermentation could be well separated from other samples. It was also evident that the content of amino acids, peptides and their analogs increased rapidly from 18 to 32 days of fermentation and then remained in dynamic equilibrium. The results also indicated that the metabolites of the Jiupei changed gradually with fermentation from 4 to 18 and 32 to 220 days. The faster change in metabolites from 18 to 32 days may be due to the higher metabolic activity of microorganisms during this period (Figure 6C).

Subsequently, six samples of Jiupei fermented for 18, 24, 32, 100, 130, and 220 days were selected for differential metabolite analysis with the control group (0 days). At JZ_18 *VS* JZ_0, we identified 115 DEMs (up-regulated: 79, down-regulated: 36), mainly lipids and lipid-like molecules (30.43%) and organic acids and derivatives (11.30%). At JZ_24 *VS* JZ_0, we identified 168 DEMs (up-regulated: 117, down-regulated: 51), mainly organic acids and derivatives (32.75%) and lipids and lipid-like molecules (18.45%). Compared with JZ_18 *VS* JZ_0, the number of amino acids, peptides and analogs increased by 24 and the number of organic acids and derivatives increased by 6 in the DEMs. At JZ_32 *VS* JZ_0, we identified 255 DEMs (up-regulated: 196, down-regulated: 59), mainly organic acids and derivatives (27.84%) and lipids and lipid-like molecules (23.92%). Compared with JZ_24 *VS* JZ_0, the number of amino acids, peptides and analogs increased by 14 and the number of sugar substances and derivatives, organic acids and derivatives increased by 4 and 5 in the DEMs, respectively. We identified 366 (up-regulated: 281, down-regulated: 85), 317 (up-regulated: 226, down-regulated: 91) and 329 (up-regulated: 241, down-regulated: 88) DEMs in JZ_100 *VS* JZ_0, JZ_130 *VS* JZ_0 and JZ_220 *VS* JZ_0 were differentially expressed, correspondingly. The metabolites of the differences were mainly organic acids and derivatives and lipids and lipid-like molecules.

Different metabolites detected in samples with different fermentation times mainly include sugars and their derivatives (sucrose, fructose, sucrose, lactose, ribose, mannose, and glucose, etc.), organic acids (palmitic acid, linoleic acid, 2-hydroxy-4-methylvaleric acid, etc.), amino acids, peptides and their analogs (norleucine, isoleucine, ornithine, protein serine, etc.). In conclusion, the relative content of sugars in the Jiupei decreased significantly at 18–32 days of fermentation and then remained dynamically stable, while organic acids and derivatives showed an upward trend throughout the fermentation process, probably due to the strong metabolic activity of microorganisms at 18–32 days, which required large amounts of sugars to meet their growth and reproduction, while the oxygen content in the cellar gradually decreased as fermentation progressed and bacteria began to produce acid by anaerobic fermentation, also indicating that the formation of flavor is a long process.

#### 3.5.4. Analysis of differential metabolic pathways

To further understand the formation mechanism of metabolites during fermentation, metabolic pathways were analyzed. Differential Abundance Score (DAS) is a pathway-based analysis of metabolic changes, which can visualize the up- and down-regulation of metabolic pathways as a whole and the type of metabolism they belong to. As shown in Figure 6E, DAS analysis of the KEGG pathway showed that 17 pathways in JZ_18 *VS* JZ_0, all of which showed up-regulation and were mainly divided into translation, nucleotide metabolism, membrane transport, lipid metabolism, carbohydrate metabolism and amino acid metabolism (please refer to Supplementary Figure S5 for the results of metabolic pathway analysis of other samples). In general, the metabolites of the 18–32 days Jiupei changed rapidly and the metabolic pathways were highly variable, while the metabolites changed slowly and the metabolic pathways did not change significantly in the later stages of fermentation. Next, we performed RefSeq’s pathway database search and metabolic pathway analysis. As shown in Figure 6F, the results of pathway analysis at JZ_18 vs. JZ_0 showed that a total of 20 pathways were obtained, and screening of key metabolic pathways revealed four key metabolic pathways, including purine metabolism, niacin and nicotinamide metabolism, alanine, aspartate and glutamate metabolism, and cysteine and methionine metabolism. In general, the key metabolic pathways changed at 18–32 days of fermentation, showing an increase in the number of amino acid-related metabolic pathways as fermentation progressed, and no significant changes in the key metabolic pathways at 100–220 days of fermentation, indicating that amino acid metabolism was vigorous at 18–32 days of fermentation and slow changes in metabolites in the wine grains at the later stages of fermentation, consistent with the above The trend of the relative content of amino acids, peptides and their analogs with fermentation time is consistent with the above.

## 4. Discussion

For thousands of years, humans have been optimizing conditions including temperature, humidity and salinity to promote the growth of certain microbial communities and have obtained a variety of fermented foods such as cheese, alcoholic beverages, sourdough bread, soy sauce and vinegar (Pang et al., 2018). Although most modern fermented foods are inoculated with specific starter cultures, it is widely believed that the native microorganisms in traditional spontaneous fermentation add to the aroma of these foods (Petruzzi et al., 2017). Since Daqu preparation and alcohol fermentation are carried out under semi-controlled conditions, their unique ecology and production processes have been well enriched with specific microbiota through long and repeated practice (Pu et al., 2021). Our results showed that the relative abundance of *Lactobacillus* increased considerably in the samples as fermentation proceeded and became the dominant bacteria during the long fermentation of the Luxiang-flavor Baijiu Jiupei. *Lactobacillus* are mostly parthenogenic anaerobic bacteria or anaerobic bacteria, which are the dominant group in the production of many traditional fermented foods and have important biological structure regulation functions (Slattery et al., 2019; Reuben et al., 2020). They can use carbohydrates from raw materials to produce lactic acid and other substances (ethanol, acetic acid, carbon dioxide, etc.) to improve the flavor and storage life of foods (Lynch et al., 2018; Touret et al., 2018). The *Lactobacillus* use sugar to produce lactic acid and acetic acid, which can react with ethanol to produce ethyl lactate and ethyl acetate, of which ethyl acetate is an important flavor substance in Baijiu (Tian et al., 2020). Meanwhile, *Lactobacillus* can produce antagonistic substances, such as bacteriocins, and compete with other microorganisms for substrates and affect the growth of other microorganisms (Aarti et al., 2016). Through the amplicon sequencing technology, this study found that the relative abundance of *Lactobacillus* in fermented Jiupei gradually increased with the fermentation time, and became an absolutely dominant group in the later stage of fermentation. The demise of microorganisms that are not adapted to the brewing environment and some potential pathogenic microorganisms during the fermentation process can be regarded as the “self-purification” of the lactic acid bacteria community in the brewing process. This phenomenon has been reported in some open multi-strain mixed fermentation processes. The microbial community of the brewing bacteria gradually stabilizes and simplifies through the environmental pressure changes (temperature, acid, anaerobic, osmotic pressure and ethanol) caused by metabolic activities (Wang et al., 2015). In a word, various *Lactobacillus* occupy an absolute dominant position in the late stage of brewing and fermentation of Luzhou-flavor baijiu, which affects the flavor and quality of Luzhou-flavor baijiu.

The microbial proteins identified in the long fermentation process of fermented Jiupei were derived from *Lactobacillus jinshani*. The content of *Lactobacillus jinshani* changed significantly at the 18th day of fermentation, and the organic acids and their derivatives were the most species determined by metabolomics, accounting for 22.27%, which also increased from the 18th day. This indicated that *Lactobacillus jinshani* played an important role in the fermentation process of fermented Jiupei, and interestingly, *Lactobacillus* also dominated the microbial communities of other Chinese Baijius with different flavors (Liu and Miao, 2020). This may be due to the use of similar fermentation substrates (sorghum and wheat) and the purpose of the fermentation ecosystem, which is to convert starch feedstocks into ethanol and flavor compounds. Metatranscriptomic analysis showed that the metabolism of flavor compounds and their precursors by *Lactobacillus* plays a key role in Luzhou-flavor Baijiu (Du et al., 2020). *Lactobacillus* was the core functional microorganism for lactic acid accumulation during the production of soy sauce aroma Baijiu (Song et al., 2017b). In the later stage, the oxygen content in the fermented Jiupei decreased, which led to the decrease of *Lactobacillus jinshani* (Wei et al., 2021). The genus *Kazachstania* was the most abundant genus of fungi, and can also affect the fermentation of Luzhou-flavor Baijiu by interacting with other mixed fermenting microorganisms. Studies have found that *K. humilis* can inhibit the production of lactic acid by *L. acetotolerans*, and *L. acetotolerans* can promote the production of *L. acetotolerans* produces ethanol, indicating that yeast of this genus can affect the fermentation process of Luzhou-flavor Baijiu through the interaction with lactic acid bacteria (Wang et al., 2021). Prolamin was the most abundant component in all flavor-type distiller’s grains powder, accounting for about 40–45% of the total extracted protein. This is mainly due to the fact that prolamin is insoluble in water and easily soluble in 70% vol. However, during the brewing process of Baijiu, the concentration of ethanol in the environment has not reached a concentration sufficient to dissolve it, so the gliadin cannot be used, so it accumulates in the Jiupei after distillation (Jiang et al., 2020). The metaproteomics analysis of the proteins in the fermented Jiupei after dehosting showed that the proteins involved in carbohydrate transport metabolism and amino acid transport metabolism during Jiupei fermentation increased significantly on the 4th day; membrane biogenesis proteins on the 18th day significantly increased, and the increase range was greater, indicating that the gene expression and metabolism of microorganisms in the system on the 4th day of fermentation of Jiupei had changed a lot. Among them, *Lactobacillus jinshani* had the highest protein expression, and *Lactobacillus jinshani* was found to be widely present in Baijius in various producing areas of the country in the brewing system (Du et al., 2020). It has been reported that *Lactobacillus jinshani* was dominant in the late stage of fermented grains, which may be related to the better acid resistance of the bacteria (Yu et al., 2020). Therefore, *Lactobacillus jinshani* in Luxiang-flavor Baijiu plays a key role in the accumulation of Jiupei proteins and the flavor of Baijiu.

## Data availability statement

The datasets presented in this study can be found in online repositories. The names of the repository/repositories and accession number(s) can be found in the article/Supplementary material.

## Author contributions

JM and CS: designed this experimental subject. XL was involved in the analysis of the entire study and drafted the manuscript. DM, CY, and QY: performed data collection, formal analysis, and data curation. JM, SL, DM, and XL critically revised the manuscript. All authors contributed to the article and approved the submitted version.

## Conflict of interest

XL was employed by Luzhou Laojiao Group Co. Ltd. SL and JM were employed by Zhejiang Guyuelongshan Shaoxing Wine Co., Ltd.

The remaining authors declare that the research was conducted in the absence of any commercial or financial relationships that could be construed as a potential conflict of interest.

## Publisher’s note

All claims expressed in this article are solely those of the authors and do not necessarily represent those of their affiliated organizations, or those of the publisher, the editors and the reviewers. Any product that may be evaluated in this article, or claim that may be made by its manufacturer, is not guaranteed or endorsed by the publisher.
