# Supplementary material for: Microbial community succession patterns and drivers of Luxiang-flavor Jiupei during long fermentation
Source: Front Microbiol. 2023 Feb 10;14:1109719. doi: 10.3389/fmicb.2023.1109719 (PMC9950560; doi:10.3389/fmicb.2023.1109719)
Supplement: Supplementary file 1 [file Data_Sheet_1.docx]

**Figure S1. Analysis of protein principal components in the fermentation process of Jiupei.**


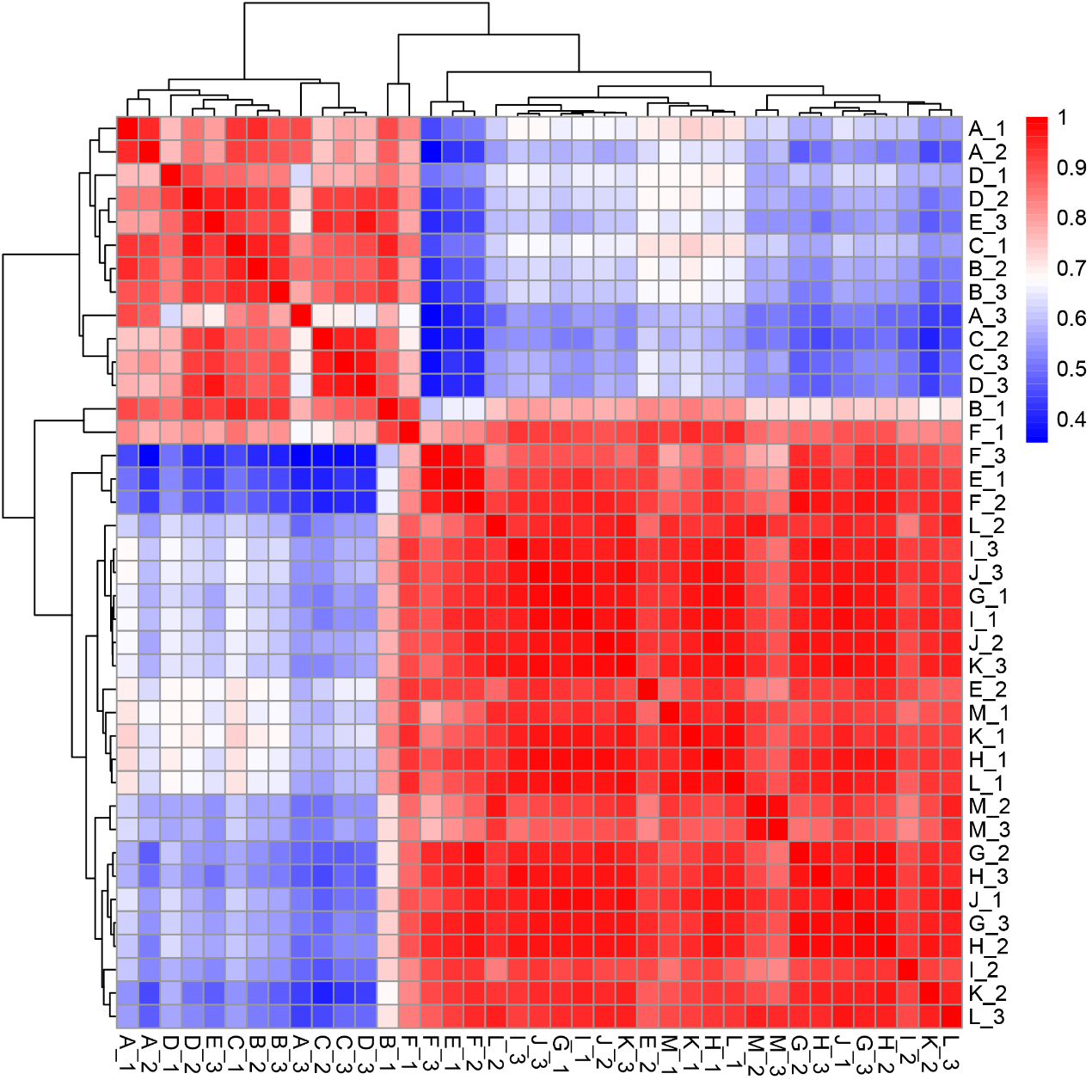


**Figure S2. Heatmap of sample correlations.**


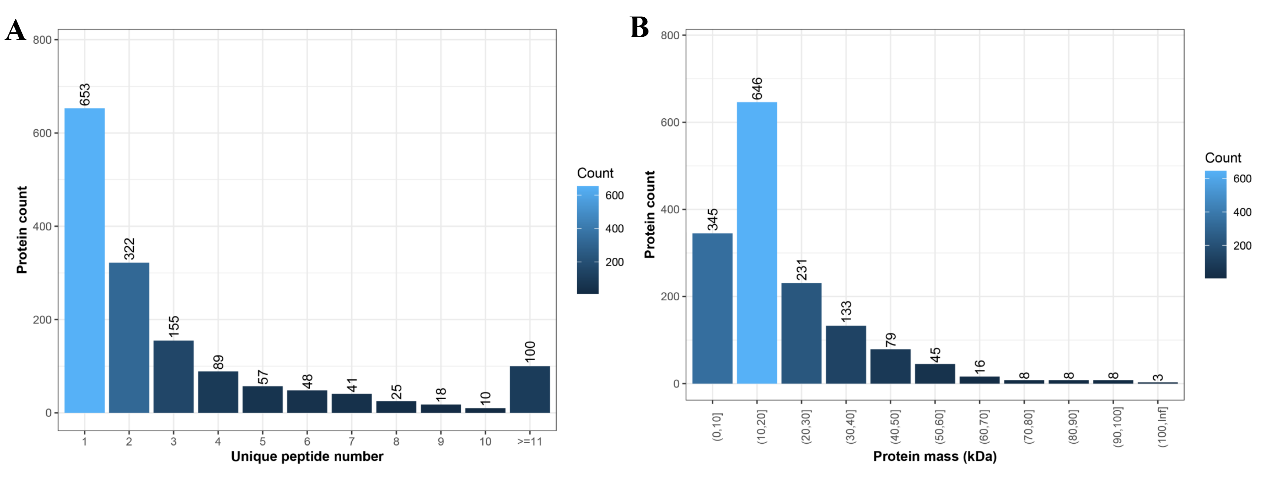


**Figure S3. Unique peptide distribution and protein mass distribution.**


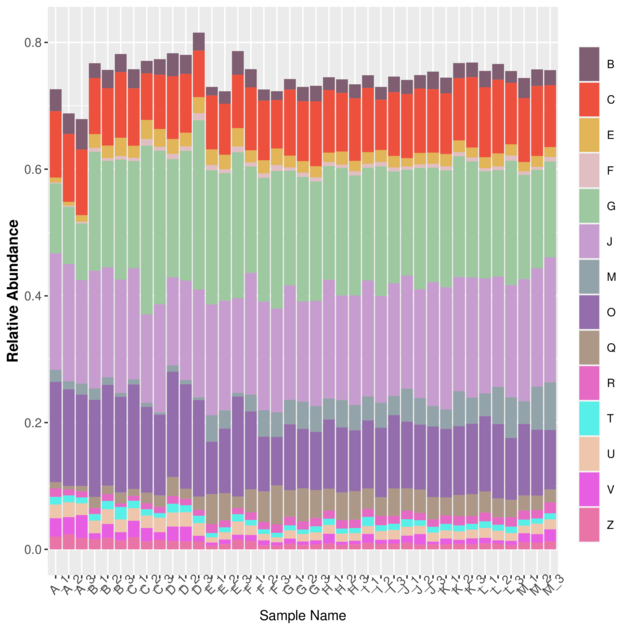


**Figure S4. Jiupei NOG functional composition analysis.** B: chromatin structure and dynamics; C: energy production and conversion; E: amino acid transport and metabolism; F: nucleotide transport and metabolism; G: carbohydrate transport and metabolism; J: translation, ribosomal structure and biogenesis; M: cell wall/membrane/envelope biogenesis; O: posttranslational modification, protein turnover, chaperones; Q: secondary metabolites biosynthesis, transport and catabolism; R: function unknown; T: signal transduction mechanisms; U: intracellular trafficking, secretion, and vesicular transport; V: defense mechanisms; Z: cytoskeleton.


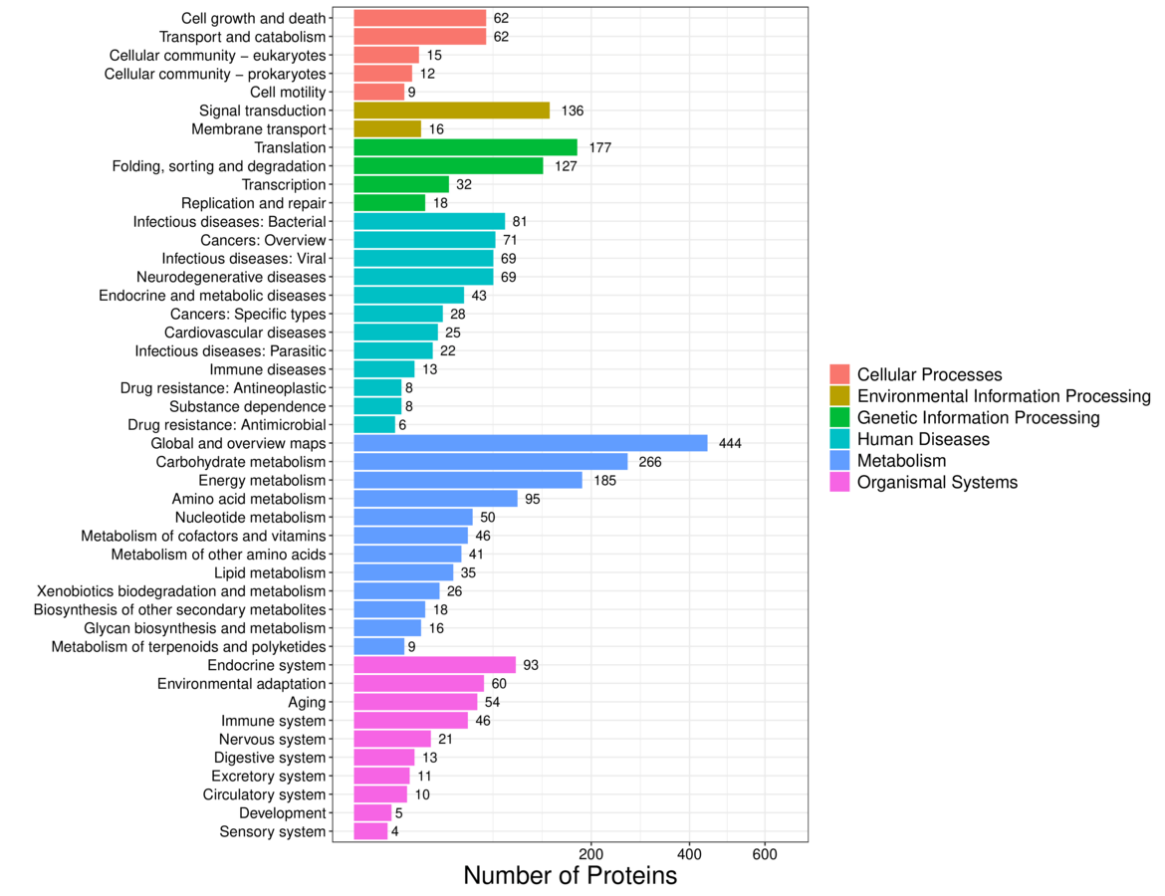


**Figure S5. Bar plot of pathway annotation**

**
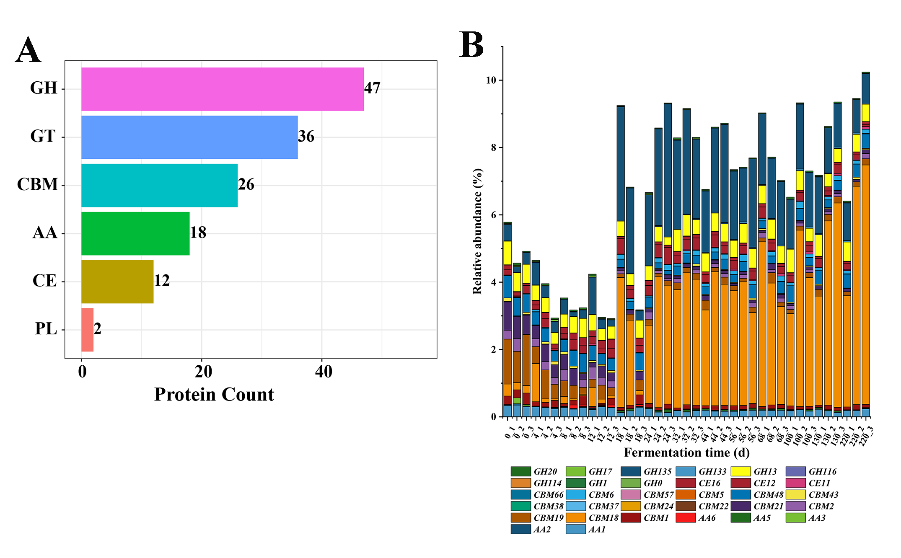
**

**Figure S6. CAZy functional annotation analysis.** (A) CAZy annotation classification. (B) Stacked histogram of the relative abundance of CAZy functions during long fermentation.


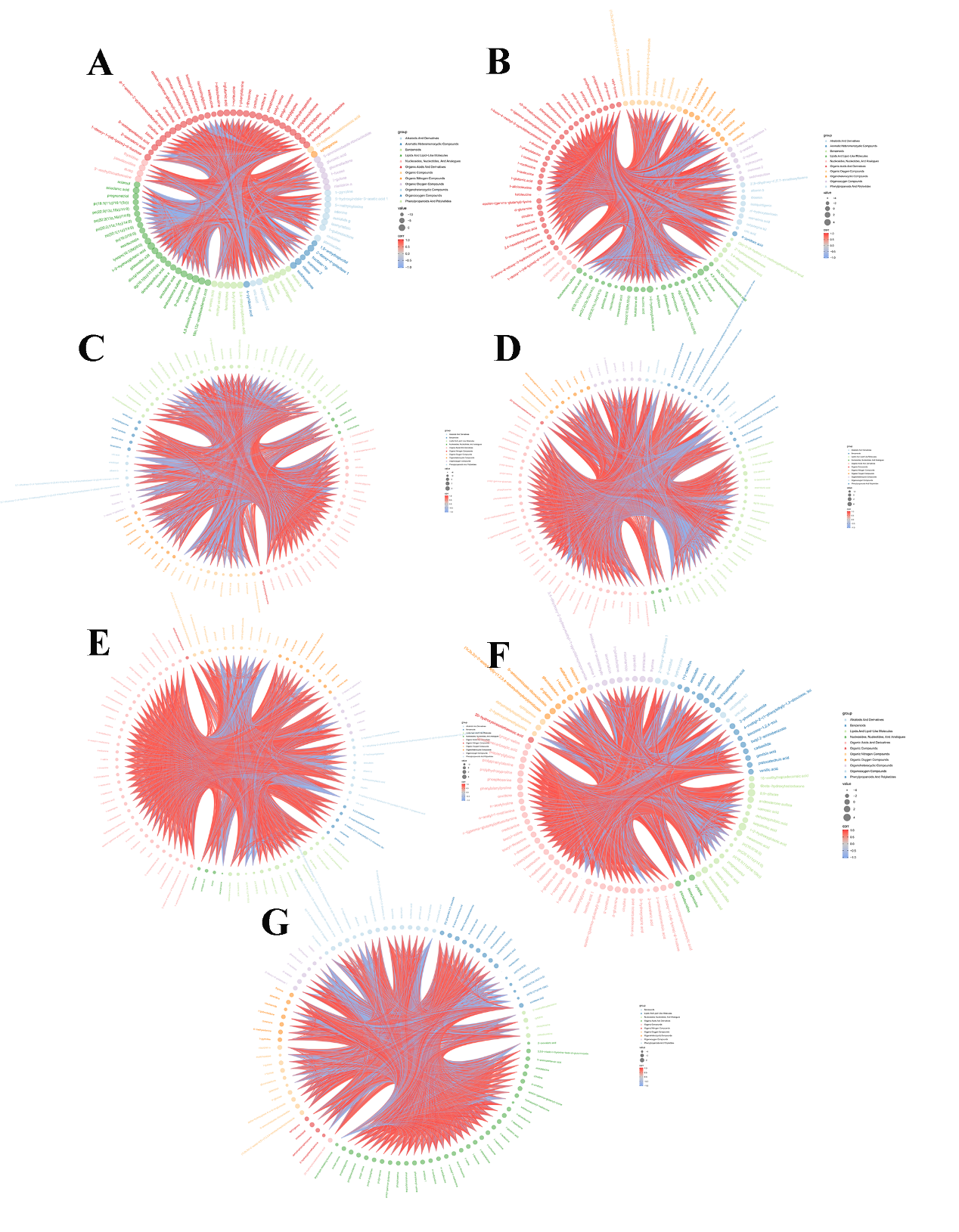


**Figure S7. Differential metabolite analysis.**


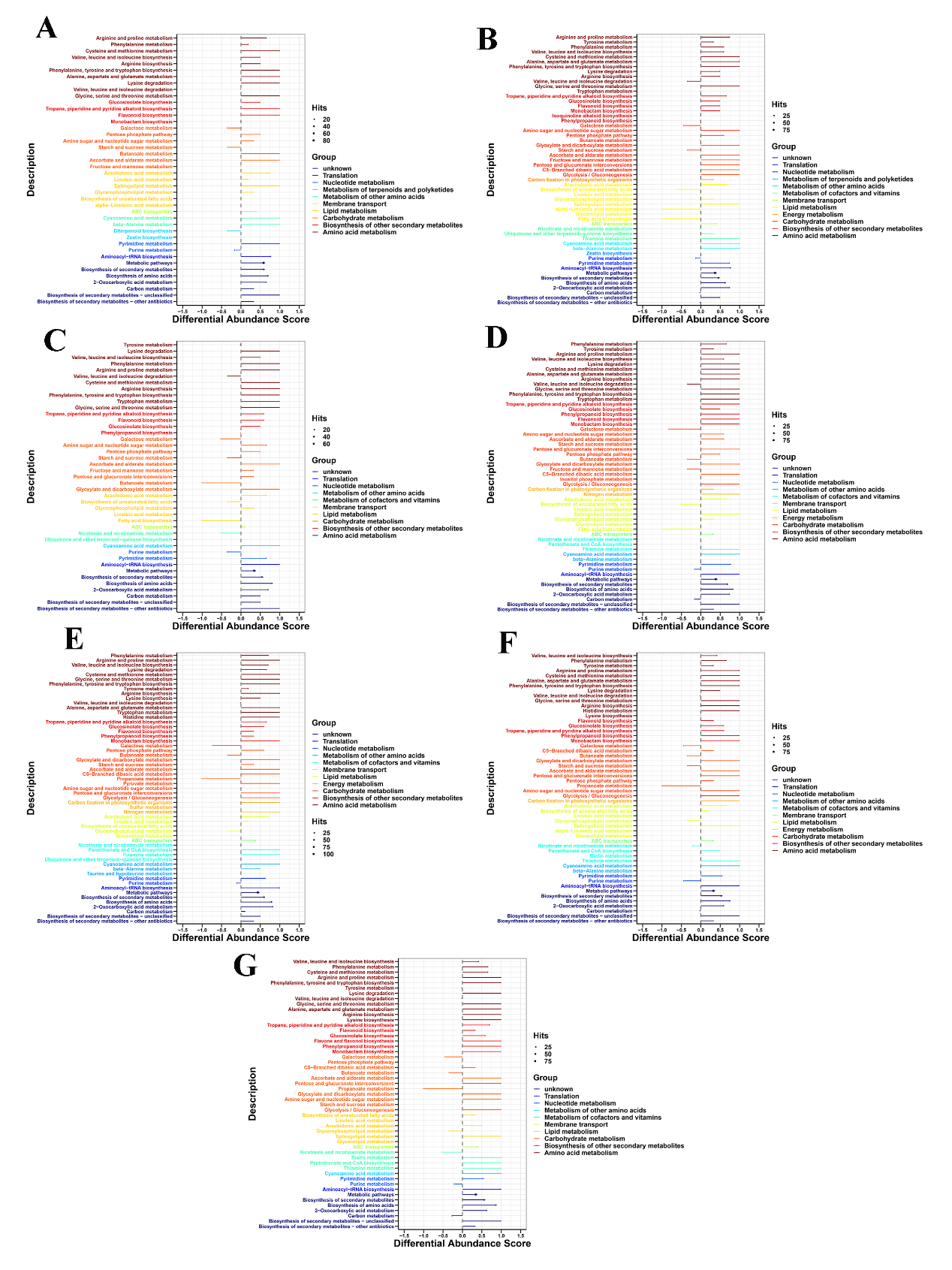


**Figure S8. KEGG pathway analysis for differential metabolites.**
