# Supplementary material for: Microbial community succession patterns and drivers of Luxiang-flavor Jiupei during long fermentation
Source: Front Microbiol. 2023 Feb 10;14:1109719. doi: 10.3389/fmicb.2023.1109719 (PMC9950560; doi:10.3389/fmicb.2023.1109719)
Supplement: Supplementary file 2 [file Data_Sheet_2.docx]

Table S1 Primers used in this experiment

|  | Forward Primers sequences | | | Reverse primers sequences |
| --- | --- | --- | --- | --- |
| 16S | | | 5Phos/GCATC+barcode sequence+AGRGTTYGATYMTGGCTCAG | 5Phos/GCATC+barcode sequence+RGYTACCTTGTTACGACTT |
| ITS | | TCCGTAGGTGAACCTGCGG | | TCCTCCGCTTATTGATATGC |

Table S2 Overview of the identification of each sample

| Sample | Total spectra | Identified spectra | Identified peptides | Quantified peptides | Identified proteins |
| --- | --- | --- | --- | --- | --- |
| A _1 | 44780 | 2694 | 2239 | 2176 | 915 |
| A_2 | 42925 | 2112 | 1817 | 1777 | 841 |
| A_3 | 44436 | 2409 | 2018 | 1970 | 858 |
| B_1 | 45591 | 3688 | 3015 | 2940 | 1039 |
| B_2 | 43333 | 2617 | 2205 | 2160 | 934 |
| B_3 | 43545 | 2600 | 2208 | 2146 | 949 |
| C_1 | 45148 | 2698 | 2275 | 2234 | 927 |
| C_2 | 44443 | 2737 | 2297 | 2235 | 960 |
| C_3 | 43388 | 2513 | 2134 | 2085 | 888 |
| D_1 | 41767 | 2793 | 2301 | 2223 | 915 |
| D_2 | 45473 | 2849 | 2380 | 2317 | 978 |
| D_3 | 43926 | 2631 | 2203 | 2144 | 899 |
| E_1 | 48485 | 5468 | 4233 | 4112 | 1156 |
| E_2 | 45851 | 4649 | 3694 | 3597 | 1100 |
| E_3 | 46018 | 2680 | 2228 | 2173 | 916 |
| F_1 | 46486 | 3974 | 3166 | 3084 | 1050 |
| F_2 | 45848 | 5298 | 4125 | 3995 | 1109 |
| F_3 | 46129 | 5494 | 4256 | 4134 | 1136 |
| G_1 | 47765 | 4583 | 3610 | 3520 | 1103 |
| G_2 | 44391 | 4819 | 3804 | 3691 | 1099 |
| G_3 | 42955 | 4796 | 3829 | 3725 | 1121 |
| H_1 | 47783 | 4663 | 3677 | 3583 | 1129 |
| H_2 | 46085 | 4920 | 3873 | 3773 | 1110 |
| H_3 | 42907 | 4731 | 3725 | 3621 | 1069 |
| I_1 | 48228 | 4814 | 3731 | 3628 | 1099 |
| I_2 | 44112 | 4544 | 3571 | 3488 | 1033 |
| I_3 | 44017 | 4397 | 3490 | 3390 | 1040 |
| J_1 | 45992 | 4693 | 3654 | 3562 | 1082 |
| J_2 | 46310 | 4734 | 3735 | 3650 | 1093 |
| J_3 | 45232 | 4567 | 3612 | 3516 | 1080 |
| K_1 | 45666 | 4692 | 3666 | 3558 | 1110 |
| K_2 | 45583 | 4759 | 3709 | 3622 | 1058 |
| K_3 | 43579 | 4600 | 3614 | 3521 | 1080 |
| L_1 | 45600 | 4585 | 3600 | 3510 | 1093 |
| L_2 | 45548 | 4321 | 3411 | 3336 | 1039 |
| L_3 | 42853 | 4567 | 3586 | 3496 | 1043 |
| M_1 | 47311 | 4647 | 3598 | 3498 | 1095 |
| M_2 | 43532 | 4122 | 3242 | 3163 | 1011 |
| M_3 | 49309 | 4231 | 3291 | 3219 | 1039 |

Table S3 Statistics of species classification annotation

| Item | Number | Percentage (%) |
| --- | --- | --- |
| Kingdom | 1431 | 93.83 |
| Phylum | 1365 | 89.51 |
| Class | 1297 | 85.05 |
| Order | 1266 | 83.02 |
| Family | 1086 | 71.21 |
| Genus | 886 | 58.10 |
| Species | 709 | 46.49 |

Table S4 Statistics of function database annotation

| Item | Number | Percentage (%) |
| --- | --- | --- |
| GO | 1257 | 82.59 |
| GO Biological_Process | 1140 | 74.91 |
| GO Molecular_Function | 1212 | 79.64 |
| GO Cellular_Component | 794 | 52.17 |
| KEGG | 1117 | 73.39 |
| KOG | 1009 | 66.29 |
| CAZy | 141 | 9.26 |
| Swissprot | 1314 | 86.33 |
